# Combining prognostic nutritional index (PNI) and controlling nutritional status (CONUT) score as a valuable prognostic factor for overall survival in patients with stage I–III colorectal cancer

**DOI:** 10.3389/fonc.2023.1026824

**Published:** 2023-01-30

**Authors:** Harin Kim, Dong-Min Shin, Jae-Hoon Lee, Eun-Suk Cho, Hye Sun Lee, Su-Jin Shin, Eun Jung Park, Seung Hyuk Baik, Kang Young Lee, Jeonghyun Kang

**Affiliations:** ^1^ Yonsei University College of Medicine, Seoul, Republic of Korea; ^2^ Department of Surgery, Gangnam Severance Hospital, Yonsei University College of Medicine, Seoul, Republic of Korea; ^3^ Department of Nuclear Medicine, Gangnam Severance Hospital, Yonsei University College of Medicine, Seoul, Republic of Korea; ^4^ Department of Radiology, Gangnam Severance Hospital, Yonsei University College of Medicine, Seoul, Republic of Korea; ^5^ Biostatistics Collaboration Unit, Yonsei University College of Medicine, Seoul, Republic of Korea; ^6^ Department of Pathology, Gangnam Severance Hospital, Yonsei University College of Medicine, Seoul, Republic of Korea; ^7^ Department of Surgery, Severance Hospital, Yonsei University College of Medicine, Seoul, Republic of Korea

**Keywords:** prognostic nutritional index (PNI), controlling nutritional status (CONUT) score, colorectal cancer, prognostic factor, inflammatory marker

## Abstract

**Background and aims:**

This study compared the prognostic significance of various nutritional and inflammatory indicators such as neutrophil-to-lymphocyte ratio, lymphocyte-to-monocyte ratio and platelet-to-lymphocyte ratio, prognostic nutritional index, and controlling nutritional status score. In addition, we aimed to establish a more accurate prognostic indicator.

**Methods:**

We retrospectively evaluated 1112 patients with stage I–III colorectal cancer between January 2004 and April 2014. The controlling nutritional status scores were classified as low (0–1), intermediate (2–4), and high (5–12) scores. The cut-off values for prognostic nutritional index and inflammatory markers were calculated using the X-tile program. P-CONUT, a combination of prognostic nutritional index and the controlling nutritional status score, was suggested. The integrated areas under the curve were then compared.

**Results:**

The multivariable analysis showed that prognostic nutritional index was an independent prognostic factor for overall survival, whereas the controlling nutritional status score, neutrophil-to-lymphocyte ratio, lymphocyte-to-monocyte ratio, and platelet-to-lymphocyte ratio were not. The patients were divided into three P-CONUT groups as follows: G1, controlling nutritional status (0–4) and high prognostic nutritional index; G2, controlling nutritional status (0–4) and low prognostic nutritional index; and G3, controlling nutritional status (5–12) and low prognostic nutritional index. There were significant survival differences between the P-CONUT groups (5-year overall survival of G1, G2, and G3 were 91.7%, 81.2%, and 64.1%, respectively; *p* < 0.0001). The integrated areas under the curve of P-CONUT (0.610, CI: 0.578–0.642) was superior to those of the controlling nutritional status score alone (bootstrap integrated areas under the curve mean difference=0.050; 95% CI=0.022–0.079) and prognostic nutritional index alone (bootstrap integrated areas under the curve mean difference=0.012; 95% CI=0.001–0.025).

**Conclusion:**

Prognostic effect of P-CONUT may be better than inflammatory markers such as neutrophil-to-lymphocyte ratio, lymphocyte-to-monocyte ratio and platelet-to-lymphocyte ratio. Thus, it could be used as a reliable nutritional risk stratification tool in patients with colorectal cancer.

## Introduction

More than 1.9 million people worldwide are diagnosed with colorectal cancer (CRC), which ranks third for incidence and second for mortality worldwide (1). In particular, the CRC incidence rate is high in South Korea, with approximately 44.5 cases per 100,000 persons ranking second highest worldwide in 2018 (2). Although the mortality rate is decreasing due to screening and improved treatments, many studies have been performed to reveal the tumor-related molecular pathways and host-related biomarkers involved in CRC (3, 4).

As host-related factors are associated with postoperative patient outcomes, many investigators have identified the prognostic predictability of nutritional and inflammatory markers in patients with CRC including the Glasgow prognostic score (GPS), the controlling nutritional status (CONUT) score, and the prognostic nutritional index (PNI) (5, 6). Nutritional markers are regarded as important pre-surgical prognostic factors because malnutrition is associated with wound healing delay, muscle weakness, immune dysfunction, infection, and further postoperative complications, leading to increased mortality (7, 8). Inflammation is also correlated with cancer progression, and several studies have identified the molecular pathways of cancer-related inflammation (9, 10).

The CONUT score, calculated from the blood cholesterol level, lymphocyte count, and serum albumin level, reflects the nutritional and immune state of the patient (11). PNI, which is derived from the serum albumin level and lymphocyte count, also reflects the nutritional and immune states of the patient (12). Despite considering the similar serum-based markers, the prognostic efficacies of the CONUT score and PNI showed some discordant results in patients with CRC, and a more reliable indicator is required for the generalized adoption of these nutritional indices.

Preoperative or postoperative decision making in patients with CRC is mainly based on the clinical or pathologic TNM staging. After confirming of CRC using colonoscopic or sigmoidoscopic biopsy, abdominopelvic computed tomography (CT) or chest CT were routinely performed to check distant metastasis or local disease extent. Treatment modality of clinical stage IV cancer is not same as the early-stage cancer patients. In addition, pathologic TNM staging is main treatment indicator especially for postoperative chemotherapy receipt. Nevertheless, survival outcome even in same stage is diverse and survival paradox such as better survival outcomes in some early stage III patients than advanced stage II patients is also demonstrated, thus we may need additional prognosticator than TNM stage (13–15).

Therefore, we investigated the prognostic impact of inflammatory and nutritional markers and compared their predictive efficacies. We also tried to establish a more valuable indicator of nutritional status to predict long-term survival outcomes in patients with CRC.

## Material and methods

### Study population

This was a retrospective cohort study with I–III CRC patients at the Gangnam Severance Hospital, Yonsei University College of Medicine, between January 2004 and April 2014. Initially, we selected 1751 patients who underwent surgical resections during this period. The patients with histologically defined neuroendocrine or gastrointestinal stromal tumors (n=115); appendix or anal cancers (n=19); tumor stage 0, IV or no information of tumor stage (n=225); hereditary nonpolyposis CRC or familial adenomatous polyposis-associated cancers (n=7); inflammatory bowel disease-associated cancers (n=3); and double-primary cancer or synchronous cancers (n=18) and those undergoing emergency surgeries (n=4) and preoperative chemoradiotherapy or radiotherapy (n=117) were excluded. Patients with no available CONUT data or blood testing results within 1 month prior to surgery (n=5) were also excluded from our study. Finally, 1112 patients were included in our study. The details of the inclusion criteria are presented in **Supplementary Figure S1**.

The study protocol adhered to the ethical standards of the institutional and/or national research committees and the 1964 Helsinki Declaration and its later amendments. The Institutional Review Board of Gangnam Severance Hospital approved this study (3–2020–0410). The requirement for written informed consent was waived owing to the retrospective study design.

### Calculations of CONUT score and PNI

The CONUT score was calculated using serum albumin (g/dL), total lymphocyte (count/mm^3^), and total cholesterol (mg/dL) levels (**Supplementary Table S1**). In our study, the patients were divided into low (0–1), intermediate (2–4), and high (5–12) CONUT score groups as previously described (5). PNI was calculated as 10 × serum albumin (g/dL) + 0.005 × total peripheral lymphocyte count (μL).

### Defining the cut-off values of PNI, NLR, LMR, and PLR

The neutrophil-to-lymphocyte ratio (NLR) was calculated by dividing the absolute neutrophil count by the absolute lymphocyte count (ALC). The lymphocyte-to-monocyte ratio (LMR) was calculated by dividing the ALC by the absolute monocyte count. The platelet-to-lymphocyte ratio (PLR) was calculated by dividing the absolute platelet count by the ALC. Given that the mean inflammatory marker values vary greatly with the type and stage of the cancer (16), we defined our own cut-off values for PNI, NLR, LMR and PLR based on the X-tile program (17).

The X-tile program is an intuitive, comprehensive open-source software for cut-off selection based on traditional statistical tests. All possible divisions of the marker data were assessed, and associations were calculated at each division. For survival, the log-rank test was used, and for the means test, associations between other marker data were used. For every possible division of the population, a χ^2^ value was calculated for the optimal division. By dividing into high and low populations using the X-tile plots, we can obtain the optimal cut-off value of each nutritional or inflammatory marker (**Supplementary Figure S2**).

### P-CONUT as a new classification that combines the CONUT score and PNI

A new classification that reflects both the CONUT score and PNI was suggested and named “P-CONUT”. The patients were allocated into low-or high-PNI groups based on the cut-off values of PNI. For a more simplified classification, the CONUT scores were also divided into two groups: low-plus-intermediate group (CONUT score 0–4) versus high group (CONUT score 5–12). Thereafter, the patients were divided into three groups (P-CONUT) as follows: G1, CONUT (0–4) and high PNI; G2, CONUT (0–4) and low PNI; G3, CONUT (5–12) and low PNI. There were no patients with CONUT (5–12) and high PNI.

### Measured outcomes

The following clinical parameters were assessed retrospectively. Sex, age (<70 vs. ≥70), body mass index (<25 vs. ≥25 kg/m^2^), carcinoembryonic antigen (CEA) (<5 vs. ≥5 ng/ml), tumor location, tumor size (<5 vs. ≥5 cm), histologic grade, lymphovascular invasion (LVI), number of metastatic lymph nodes (LN), and tumor stage according to the Union for International Cancer Control TNM classification. Blood tests were performed within 1 month prior to surgery to calculate the CONUT score, PNI, NLR, LMR, and PLR.

### Follow-up

All the patients underwent surgical resections, and the specimens were histologically assessed by experienced pathologists. Postoperative follow-up was done every 3-6 months to assess tumor recurrence. Blood tests were performed at every visit. Chest and abdominopelvic CT were performed every 6–12 months. Adjuvant chemotherapy is mainly recommended for patients with high-risk stage II or stage III CRC. High risk was defined as 1) less than 12 retrieved LN, 2) preoperative obstruction or perforation, 3) LVI positive, or 4) poorly differentiated adenocarcinoma, signet-ring cell carcinoma, or mucinous carcinoma. Colonoscopy was generally recommended for patients at one, three, and five years postoperatively. All patients were followed up until October 2019 or their deaths.

### Statistical analyses

All the analyses were performed using R version 4.1.0 (R-project, Institute for Statistics and Mathematics, Vienna, Austria). The chi-squared test or Fisher’s exact test was used to compare the categorical variables. The Mann–Whitney U test was used to compare continuous variables. Overall survival (OS) was defined as the duration from date of the operation to the date of death by any cause.

The Kaplan–Meier method with the log-rank test was used to analyze the patients’ OS. Multivariable analysis with backward selection was performed to identify the independent risk factors for OS. As the CONUT score, PNI, and P-CONUT can affect each other, we evaluated three different multivariable models, by including each variable independently. The integrated area under the curve (iAUC) was used to compare the association of OS with the PNI, CONUT, and P-CONUT. Statistical significance was set at *p* < 0.05.

## Results

### Clinical characteristics

The patient characteristics are shown in **Table 1**. A total of 1112 patients with a median age of 64 years were included in this study, out of which 667 (60%) patients were men and 445 (40%) were women. Primary tumors were located in the right colon in 292 (26.3%) patients, left colon in 342 (30.8%) patients, and rectum in 478 (43%) patients. A total of 1021 (91.8%) tumors were histologically well-differentiated or moderately differentiated, whereas the other 91 (8.2%) tumors were histologically poorly differentiated or were identified to be either signet ring cell carcinoma or mucinous adenocarcinoma.

**Table 1:** Patient’s demographics.

		N (%)
Sex	Female	445 (40)
	Male	667 (60)
Age (years)	< 70	743 (66.8)
	≥ 70	369 (33.2)
BMI (kg/m^2^)	< 25	782 (70.3)
	≥ 25	330 (29.7)
CEA (ng/mL)	< 5	767 (69)
	≥ 5	290 (26.1)
	No data	99 (4.9)
Tumor location	Right colon	292 (26.3)
	Left colon	342 (30.8)
	Rectum	478 (43)
Tumor size (cm)	< 5	691 (62.1)
	≥ 5	421 (37.9)
Histologic grade	G1 & G2	1021 (91.8)
	G3, MC & SRC	91 (8.2)
LVI	Absent	751 (67.5)
	Present	216 (19.4)
	No data	145 (13)
LN numbers	< 12	184 (16.5)
	≥ 12	928 (83.5)
Stage	I	297 (26.7)
	II	367 (33)
	III	448 (40.3)
CONUT	Low (0-1)	649 (58.4)
	Intermediate (2-4)	397 (35.7)
	High (5-12)	66 (5.9)
PNI	Mean (SD)	51.28 (6.37)
NLR	Mean (SD)	2.73 (2.07)
LMR	Mean (SD)	5.40 (2.32)
PLR	Mean (SD)	170.29 (84.75)

SD, Standard deviation.

BMI, Body mass index; CEA, Carcinoembryonic antigen; MC, Mucinous carcinoma; SRC, Signet ring cell carcinoma; LVI, Lymphovascular invasion; LN, Lymph node; CONUT, Controlling nutritional status; PNI, Prognostic nutritional index; NLR, Neutrophil to lymphocyte ratio; LMR, Lymphocyte to monocyte ratio; PLR, Platelet to lymphocyte ratio.

According to the CONUT score classification, 649 (58.4%) patients were classified into the low-CONUT score group; 397 (35.7%), intermediate-CONUT score group; and 66 (5.9%), high-CONUT score group.

### Cut-off values of PNI, NLR, LMR, and PLR

The cut-off values of PNI, NLR, LMR and PLR were 50.9, 2.0, 2.91, and 185.84, respectively (**Supplementary Figure S3**). Accordingly, 477 (42.9%) and 635 (57.1%) patients were classified into the low- and high-PNI groups, respectively. The NLR cut-off value of 2.0 classified 465 (41.82%) and 647 (58.18%) patients into the low- and high- NLR groups, respectively. The LMR cut-off value of 2.91 classified 127 (11.42%) and 985 (88.58%) patients into the low- and high- LMR groups, respectively. The PLR cut-off value of 185.84 classified 767 (68.97%) and 345 (31.03%) patients into the low- and in the high-PLR groups, respectively.

### Kaplan–Meier survival curves according to the CONUT score, PNI, and P-CONUT

**Figure 1:**
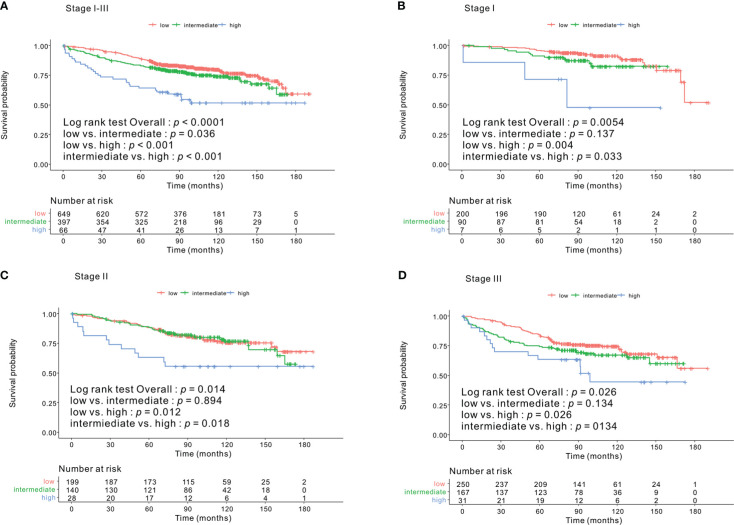
The 5-year OS rates of patients in the low-, intermediate-, and high- CONUT score groups were 89%, 83%, and 64.1% (*p* < 0.0001) **(A)**, respectively. In the tumor stage I subgroup, they were 95.5%, 89.9%, and 71.4% (*p* = 0.005) **(B)**; stage II subgroup, 87.8%, 88.4%, and 63.0% (*p* = 0.014) **(C)**; and for stage III subgroup, 83.9%, 74.2%, and 63.3% (*p* = 0.026) **(D)**, respectively. OS, overall survival; CONUT, controlling nutritional status.

**Figure 2:**
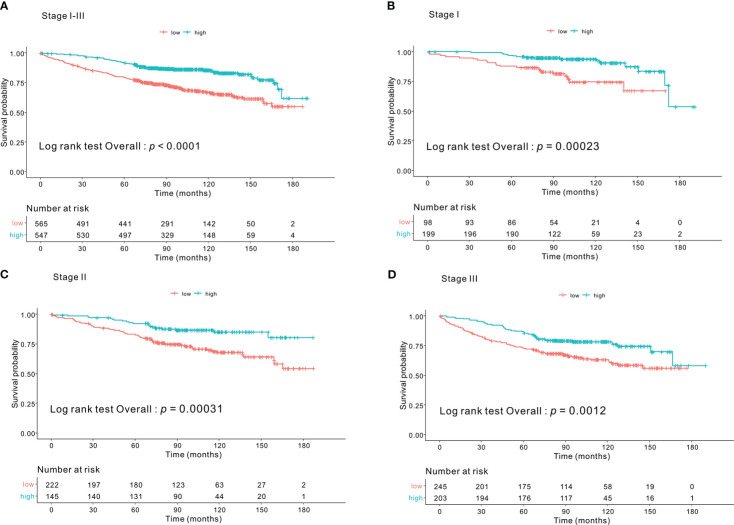
The 5-year OS rates of patients in the high- and low- PNI groups were 91.7% and 79.3% (*p* < 0.0001) **(A)**, respectively. In tumor stage I subgroup, they were 96.4% and 86.7% (*p* = 0.001) **(B)**; stage II subgroup, 92.3% and 82.6% (*p* = 0.001) **(C)**; and stage III subgroup, 86.2% and 72.8% (*p* = 0.001) **(D)**, respectively. OS, overall survival; PNI, prognostic nutritional index.

**Figure 3:**
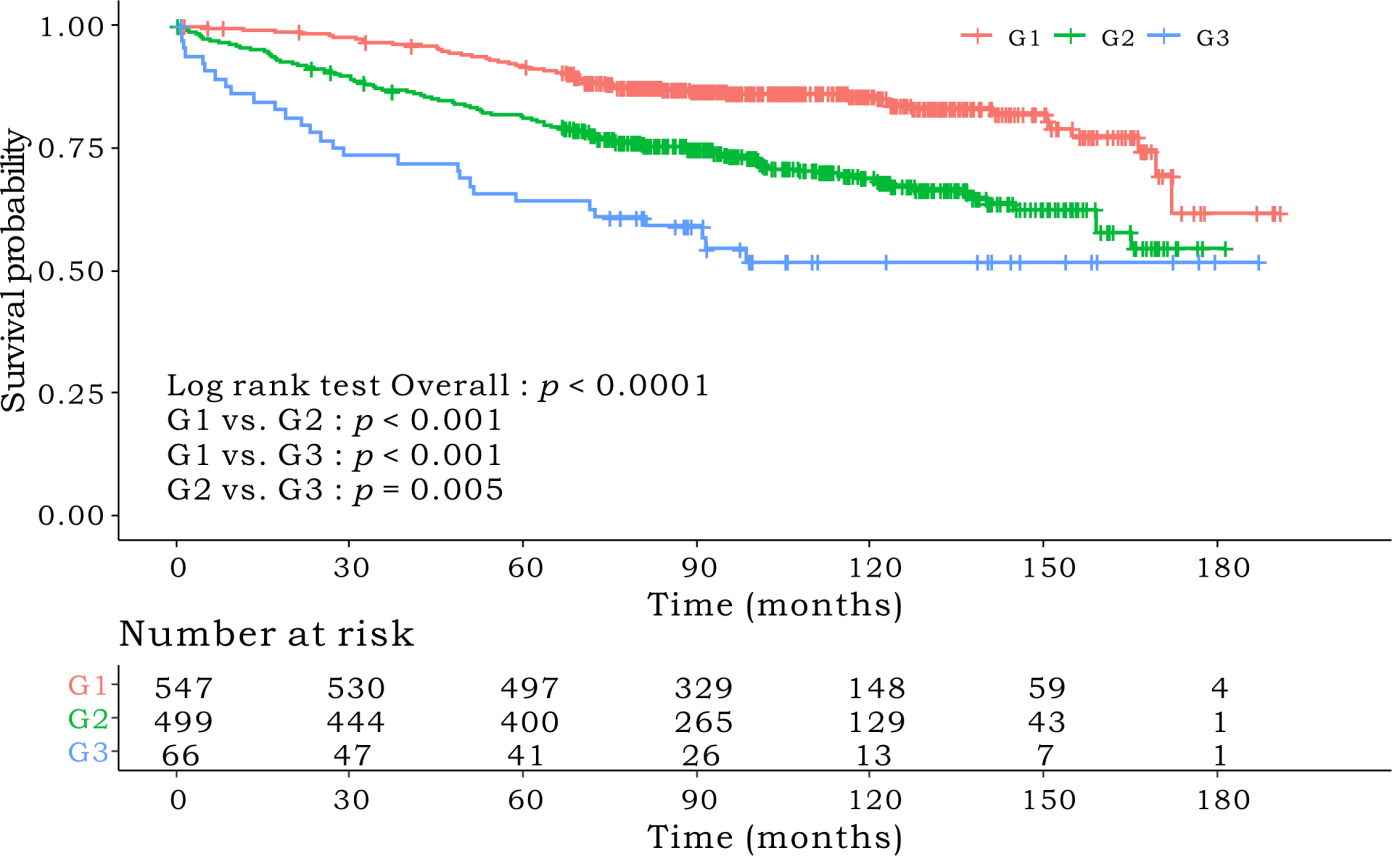
The 5-year OS rates of patients in P-CONUT G1, G2, and G3 were 91.7%, 81.2%, and 64.1% (*p* < 0.0001), respectively. OS, overall survival; P-CONUT, combination of PNI (prognostic nutritional index) and CONUT (controlling nutritional status).

Kaplan–Meier survival curves based on the CONUT scores are shown in **Figure 1**. The 5-year OS rates were 89%, 83%, and 64.1% for the low-, intermediate- and high-CONUT score groups, respectively (*p* < 0.0001). This trend was maintained in the subgroups divided into stages I, II, and III. Kaplan–Meier survival curves according to the low- and high- PNI groups are shown in **Figure 2**. The 5-year OS rates were 91.7% and 79.3% in the high- and low- PNI groups, respectively (*p* < 0.0001). On analyzing by stage, the prognosis was clearly different according to the PNI for all the stages. **Figure 3** shows the Kaplan–Meier survival curve according to P-CONUT. There were significant survival differences between the P-CONUT groups (5-year OS of G1, G2, and G3 were 91.7%, 81.2% and 64.1%, respectively; *p* < 0.0001).

### Univariable and multivariable survival analysis

In the univariable analysis, sex (*p* = 0.008), age (*p* < 0.001), CEA (*p* < 0.001), tumor size (*p* < 0.001), LVI (*p* < 0.001), LN number (*p* < 0.001), stage (stage I vs. stage II, *p* < 0.001 and stage I vs. III, *p* < 0.001), NLR (*p* < 0.001), LMR (*p* < 0.001), and PLR (*p* = 0.016) were associated with OS. In addition, the CONUT group and PNI were also identified as significant prognostic factors in the univariable analysis [low- vs. intermediate-CONUT score group (*p* = 0.041), low vs. high score group (*p* < 0.001), low- vs. high-PNI group (*p* < 0.001)] (**Table 2**).

**Table 2:** Univariable analysis associated with the overall survival (n=1112).

		Univariable analysis	
		HR (95% CI)	*p*
Sex	Female	Ref	
	Male	1.415 (1.092 – 1.833)	0.008
Age (years)	< 70	Ref	
	≥ 70	3.221 (2.521 – 4.117)	<0.001
BMI (kg/m^2^)	< 25	Ref	
	≥ 25	0.777 (0.589 – 1.025)	0.074
CEA (ng/mL)	< 5	Ref	
	≥ 5	1.708 (1.323 – 2.205)	<0.001
	No data	0.822 (0.420 – 1.611)	0.57
Tumor location	Right colon	Ref	
	Left colon	0.885 (0.655 – 1.195)	0.426
	Rectum	0.974 (0.710 – 1.335)	0.871
Tumor size (cm)	< 5	Ref	
	≥ 5	1.524 (1.195 – 1.942)	<0.001
Histologic grade	G1 & G2	Ref	
	G3, MC & SRC	1.25 (0.827 – 1.888)	0.289
LVI	Absent	Ref	
	Present	1.763 (1.317 – 2.360)	<0.001
	No data	1.493 (1.069 – 2.085)	0.018
LN numbers	< 12	Ref	
	≥ 12	0.630 (0.476 – 0.832)	0.001
Stage	I	Ref	
	II	1.918 (1.311 – 2.807)	<0.001
	III	2.685 (1.874 – 3.848)	<0.001
Chemotherapy	No	Ref	
	Yes	0.829 (0.647 – 1.063)	0.139
CONUT	Low (0-1)	Ref	
	Intermediate (2-4)	1.310 (1.010 – 1.699)	0.041
	High (5-12)	2.693 (1.801 – 4.026)	<0.001
PNI	Low	Ref	
	High	0.434 (0.334 – 0.562)	<0.001
NLR	Low	Ref	
	High	1.566 (1.211 – 2.026)	<0.001
LMR	Low	Ref	
	High	0.546 (0.393 – 0.758)	<0.001
PLR	Low	Ref	
	High	1.360 (1.057 – 1.749)	0.016
P-CONUT	G1	Ref	
	G2	2.148 (1.644 – 2.807)	<0.001
	G3	3.682 (2.412 – 5.620)	<0.001

HR, Hazard Ratio; CI, Confidence Interval.

BMI, Body mass index; CEA, Carcinoembryonic antigen; MC, Mucinous carcinoma; SRC, Signet ring cell carcinoma; LVI, Lymphovascular invasion; LN, Lymph node; CONUT, Controlling nutritional status; PNI, Prognostic nutritional index; NLR, Neutrophil to lymphocyte ratio; LMR, Lymphocyte to monocyte ratio; PLR: Platelet to lymphocyte ratio.

After adjusting for clinical and pathologic factors by multivariable analysis with backward selection, age, CEA, number of LN, stage, PNI (HR=0.603, CI: 0.455–0.800, *p* < 0.001), and P-CONUT (G1 vs. G2, HR=1.599, CI: 1.199–2.131, *p* = 0.001, G1 vs. G3, HR=2.227, CI: 1.414–3.507, *p* < 0.001) were identified as independent risk factors for OS (**Table 3**). NLR, LMR, and PLR were eliminated from the model, suggesting that these may be less predictable clinical parameters. Furthermore, the intermediate- and high-CONUT score was not correlated with OS (*p* = 0.433 and *p* = 0.125, respectively) in the multivariable analyses.

**Table 3:** Multivariable analysis associated with the overall survival (n=1112).

		Model 1 (CONUT)		Model 2 (PNI)		Model 3 (P-CONUT)	
		HR (95% CI)	*p*	HR (95% CI)	*p*	HR (95% CI)	*p*
Sex	Female	Ref		Ref		Ref	
	Male	1.258 (0.965-1.639)	0.089	1.276 (0.981-1.659)	0.068	1.281 (0.985-1.665)	0.064
Age (years)	< 70	Ref		Ref		Ref	
	≥ 70	2.988 (2.318-3.853)	<0.001	2.772 (2.149-3.577)	<0.001	2.719 (2.104-3.513)	<0.001
CEA (ng/mL)	< 5	Ref		Ref		Ref	
	≥ 5	1.356 (1.038-1.772)	0.025	1.358 (1.042-1.770)	0.023	1.339 (1.026-1.746)	0.031
	No data	1.087 (0.550-2.147)	0.808	1.103 (0.559-2.175)	0.777	1.099 (0.557-2.167)	0.785
Tumor size (cm)	< 5	Ref		Ref		Ref	
	≥ 5	1.323 (1.001-1.748)	0.049	1.248 (0.944-1.650)	0.119	1.239 (0.937-1.639)	0.132
LVI	Absent	Ref		Ref		Ref	
	Present	1.345 (0.989-1.830)	0.058	1.297 (0.951-1.768)	0.099	1.281 (0.940-1.746)	0.116
	No data	1.493 (1.069-2.085)	0.053	1.422 (1.008-2.007)	0.044	1.421(1.009-2.002)	0.044
LN numbers	< 12	Ref		Ref		Ref	
	≥ 12	0.511 (0.374-0.698)	<0.001	0.512 (0.376-0.698)	<0.001	0.504 (0.369-0.688)	<0.001
Stage	I	Ref		Ref		Ref	
	II	1.695 (1.120-2.565)	0.012	1.629 (1.075-2.468)	0.021	1.629 (1.075-2.470)	0.021
	III	2.506 (1.697- 3.701)	<0.001	2.417 (1.634-3.577)	<0.001	2.413 (1.631-3.570)	<0.001
NLR	Low	-		-		-	
	High	-		-		-	
LMR	Low	-		-		-	
	High	-		-		-	
PLR	Low	-		-		-	
	High	-		-		-	
CONUT	Low (0-1)	Ref					
	Intermediate (2-4)	1.121 (0.670-1.186)	0.433				
	High (5-12)	2.693 (1.801-4.026)	0.125				
PNI	Low			Ref			
	High			0.603 (0.455-0.800)	<0.001		
P-CONUT	G1					Ref	
	G2					1.599 (1.199-2.131)	0.001
	G3					2.227 (1.414-3.507)	<0.001

CEA, Carcinoembryonic antigen; LVI, Lymphovascular invasion; LN, Lymph node; CONUT, Controlling nutritional status; PNI, Prognostic nutritional index; NLR, Neutrophil to lymphocyte ratio; LMR, Lymphocyte to monocyte ratio; PLR, Platelet to lymphocyte ratio.

### Comparison of integrated AUC

The time-dependent receiver operating characteristic curve of P-CONUT (0.610, CI: 0.578–0.642) was superior to those of the CONUT score alone (0.560, CI: 0.528–0.590, bootstrap iAUC mean difference=0.050; 95% CI=0.022–0.079) and PNI alone (0.598, CI: 0.568–0.629, bootstrap iAUC mean difference=0.012; 95% CI=0.001–0.025) during the follow-up period (**Supplementary Figure S3**).

## Discussion

This study demonstrated that PNI is a more significant preoperative predictor of OS than the CONUT score or inflammatory serum markers for patients with stage I–III CRC who underwent surgical resections. In addition, P-CONUT, a new variable that integrates PNI and CONUT, showed superior risk stratification in predicting OS as compared to the CONUT score and PNI throughout the follow-up period. Thus, this new indicator seems to be an easy and effective way to increase the utilization of nutritional status for clinical decision-making.

As nutritional and inflammatory markers can be easily and routinely detected in clinical practice, it is important to check patients’ preoperative nutritional and inflammatory states in simple and economical ways. The CONUT score was reported to be correlated with subjective global assessment (18), and it has the advantage of being able to evaluate the nutritional status more objectively. In addition, the PNI has already been suggested as an important nutritional prognostic factor in patients with CRC (19). The PNI and CONUT scores reflect both nutritional and inflammatory statuses, as they account for both albumin and lymphocyte count. Hypoalbuminemia is common among patients with malignancies, and serum albumin level is an important prognostic factor for patients with CRC (20). Lymphocytes represent a patient’s inflammatory state. High levels of inflammatory markers are associated with poor prognosis because systemic inflammation can lead to primary tumor invasion and proliferation, angiogenesis, metastasis, and suppressed anti-tumor immunity in CRC (21).

Serum inflammatory markers such as NLR, PLR, and LMR are clinically significant prognostic markers (22, 23). Whereas, Ahiko et al. demonstrated that preoperative nutritional status is a more promising host-related prognostic factor for OS in 1880 patients with stage II and III CRC. They incorporated two nutritional indices (PNI and CONUT) and four inflammatory scores (mGPS, NLR, PLR, and C-reactive protein/albumin ratio [CAR]) in the survival analysis and showed that PNI, CONUT score, NLR, and CAR were independent prognostic factors for OS. Interestingly, nutritional indices showed better stratification performance than the other four inflammatory scores (24). Similarly, we analyzed the prognostic significance of nutritional and inflammatory markers together. Our three different multivariable analyses suggested that PNI or P-CONUT has a significant role, whereas serum inflammatory markers do not, which is in agreement with the results of Ahiko et al.

CONUT and PNI seem to play similar roles in that the items used to calculate the values (e.g., albumin and lymphocyte) were not different. Although several studies have compared the significance of nutritional markers such as the CONUT score and PNI comprehensively for predicting the survival of patients with CRC, the results are somewhat contradictory. Some studies demonstrated the CONUT score showed better prognostic significance than PNI (25–27) whereas others reported that PNI showed better stratification than the CONUT score (24, 28). Although we cannot understand the exact reason for this discordance, different cut-off values of each variable, differences in the sample size, and different disease stages might play a role. In our study, PNI was identified as a significant prognostic factor in the multivariable analysis, whereas the CONUT score was not. Furthermore, the importance of our study is that we attempted to overcome the discordance of previous results. We integrated the CONUT score and PNI and suggested a new nutritional criterion called P-CONUT and showed its superior discriminatory performance compared to these two traditional values. Our new indicator may integrate the different prognostic significance of CONUT score and PNI and overcome the limitation of applying these values in clinical practice, although this needs to be validated in further studies.

There are some limitations to our study. First, this was a single-center retrospective study, although the sample size was large. Second, since there is no universal cut-off value for PNI and inflammatory markers, we derived our own. Furthermore, regarding the CONUT score, the criteria for classification into groups varied between studies and the cut-off values of CONUT score differed, with values such as 1.5 (26) or 2 (25, 27). (**Supplementary Table S2**) Since the cut-off value may vary depending on stage, cancer type, or ethnicity, this could have influenced the result. Third, other inflammatory scores, CAR, and mGPS could not be included in our study due to the retrospective study design. Fourth, it is thought that the differences in the various clinical situations of patients can act as a difficulty in drawing conclusions. The application of adjuvant chemotherapy may also vary from person to person. Therefore, we tried to correct this part by performing multivariable analysis. However, we believe there may be limitations in this area. Finally, our study could not evaluate added value of P-CONUT in changing clinical decision, such as postoperative chemotherapy receipt or not. Rather we are in a situation where we have proposed a new combination that is slightly more accurate than the existing variables. Since the clinical usage is very important, it seems necessary to confirm it through additional research.

## Conclusion

In conclusion, our study suggested the role of PNI as a more accurate prognostic indicator than the CONUT score for patients with CRC. P-CONUT, a new classification that reflects both the CONUT score and PNI, was the most accurate. Therefore, we suggest using P-CONUT as a preoperative OS predictor for patients with stage I–III CRC.

## Data availability statement

The datasets presented in this article are not readily available because the datasets can be used after institutional review board approval owing to ethical and privacy restrictions. Requests to access the datasets should be directed to JK, ravic@naver.com.

## Ethics statement

The studies involving human participants were reviewed and approved by The Institutional Review Board of Gangnam Severance Hospital.

## Author contributions

First author: HK. Corresponding author: JK. Coauthors: D-MS, JHL, E-SC, HSL, S-JS, EJP, SHB, and KYL. All authors contributed to the article and approved the submitted version.
