# Supplementary material for: Combining prognostic nutritional index (PNI) and controlling nutritional status (CONUT) score as a valuable prognostic factor for overall survival in patients with stage I–III colorectal cancer
Source: Front Oncol. 2023 Jan 30;13:1026824. doi: 10.3389/fonc.2023.1026824 (PMC9923046; doi:10.3389/fonc.2023.1026824)
Supplement: Supplementary file 1 [file DataSheet_1.docx]

**Supplementary File**

**Supplementary Figure S1. Inclusion of patients.**


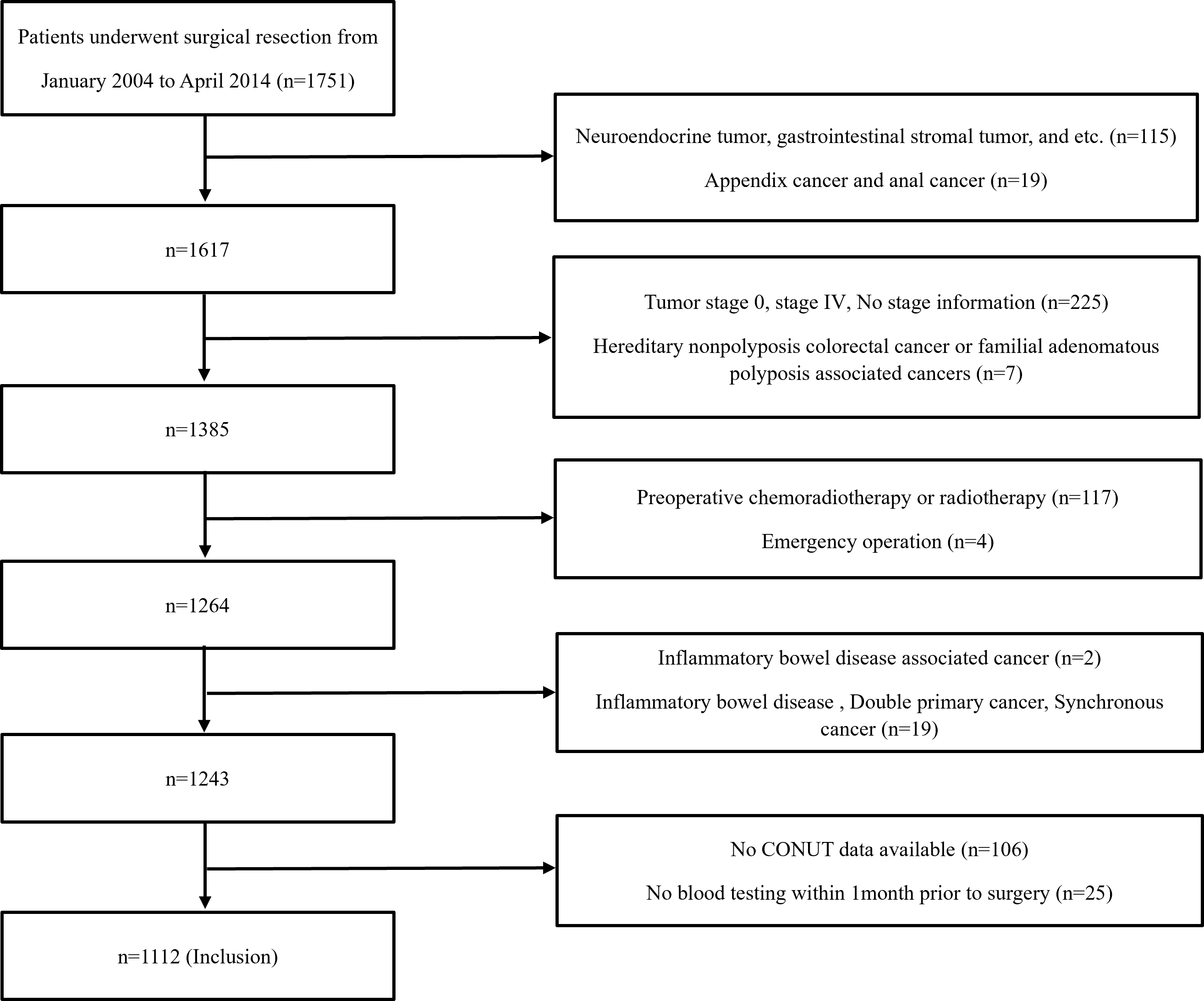


**Supplementary Table S1. Definition of CONUT score.**

| Parameters | Normal | Light | Moderate | Severe |
| --- | --- | --- | --- | --- |
| Serum albumin (g/dl) | ≥3.5 | 3.0-3.49 | 2.5-2.9 | <2.5 |
| Score | 0 | 2 | 4 | 6 |
| Total lymphocyte (count/mm^3^) | ≥1600 | 1200-1599 | 800-1199 | <800 |
| Score | 0 | 1 | 2 | 3 |
| Total cholesterol (mg/dl) | ≥180 | 140-179 | 100-139 | <100 |
| Score | 0 | 1 | 2 | 3 |
| CONUT score (total) | 0-1 | 2-4 | 5-8 | 9-12 |
| Assessment | Low | Intermediate | High | |

**Supplementary Figure S2. Defining optimal cut-off value for PNI, NLR, LMR, and PLR using X-tile program.**

| **(A) PNI** |
| --- |
| **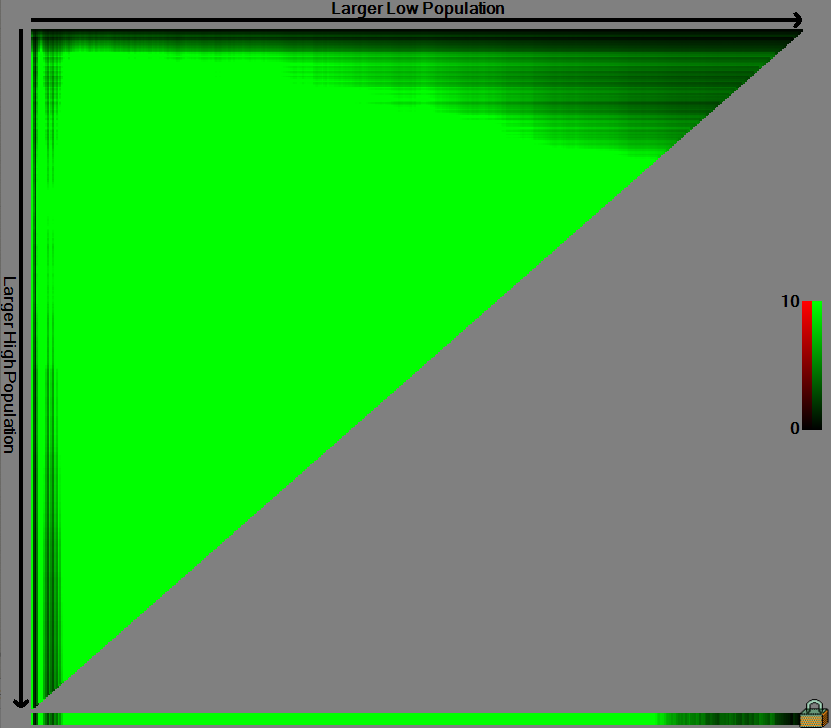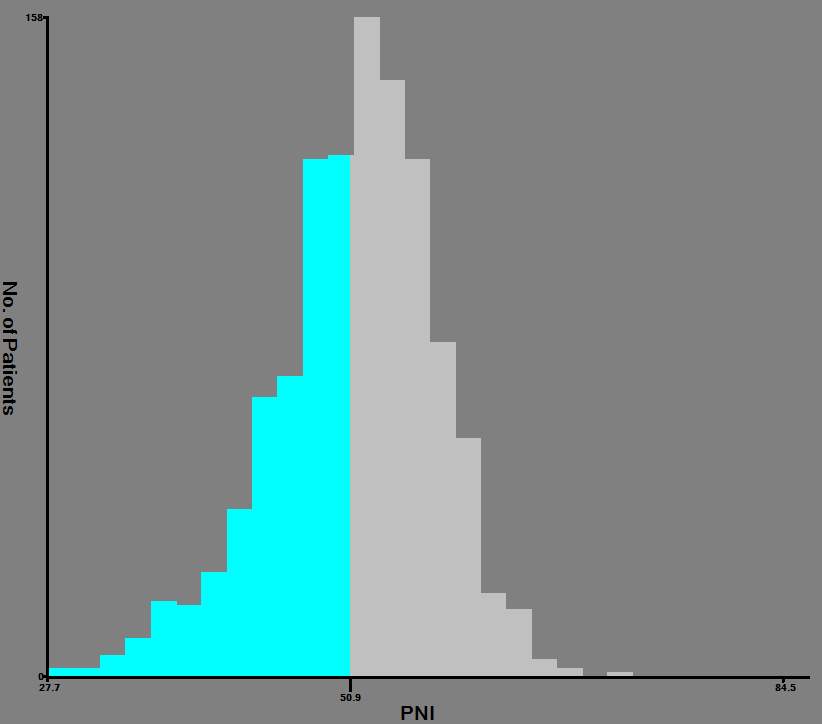** |
| **(B) NLR** |
| **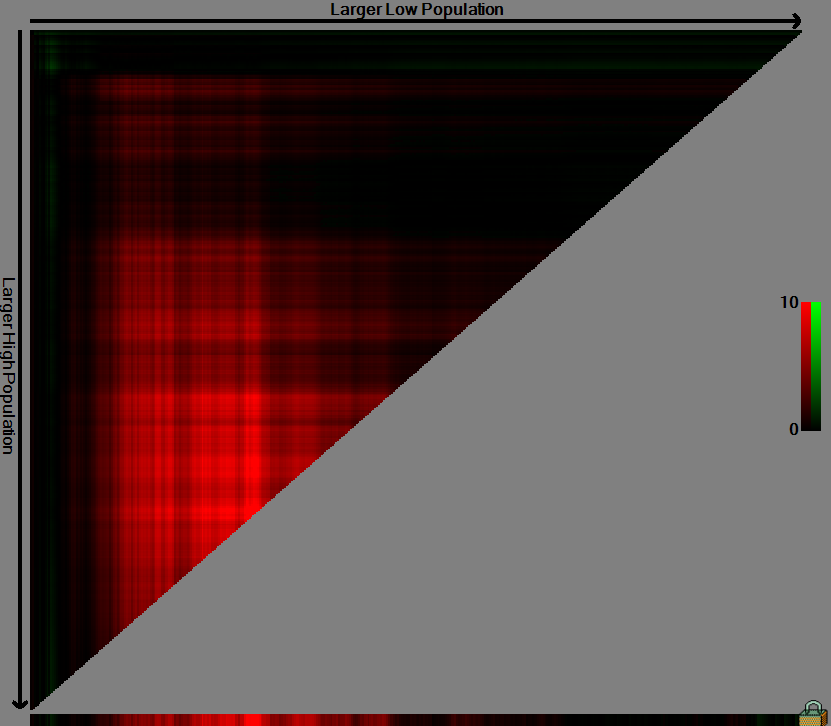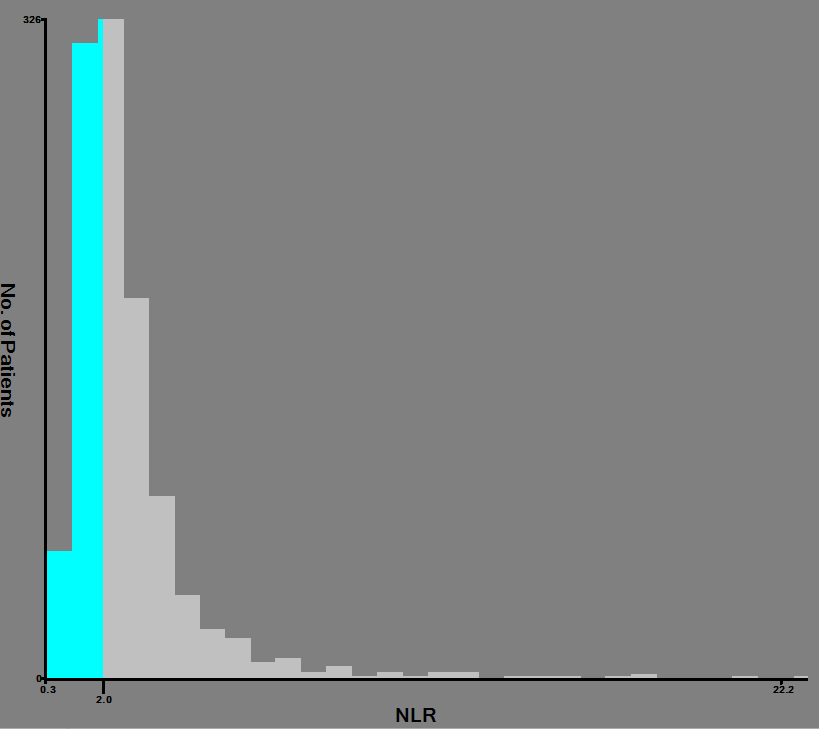** |
| **(C) LMR** |
| **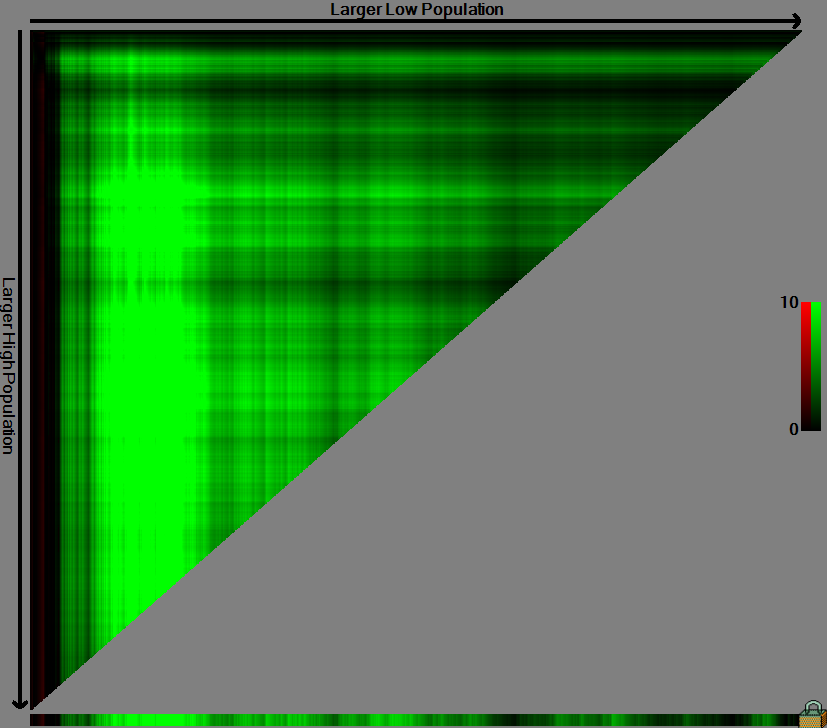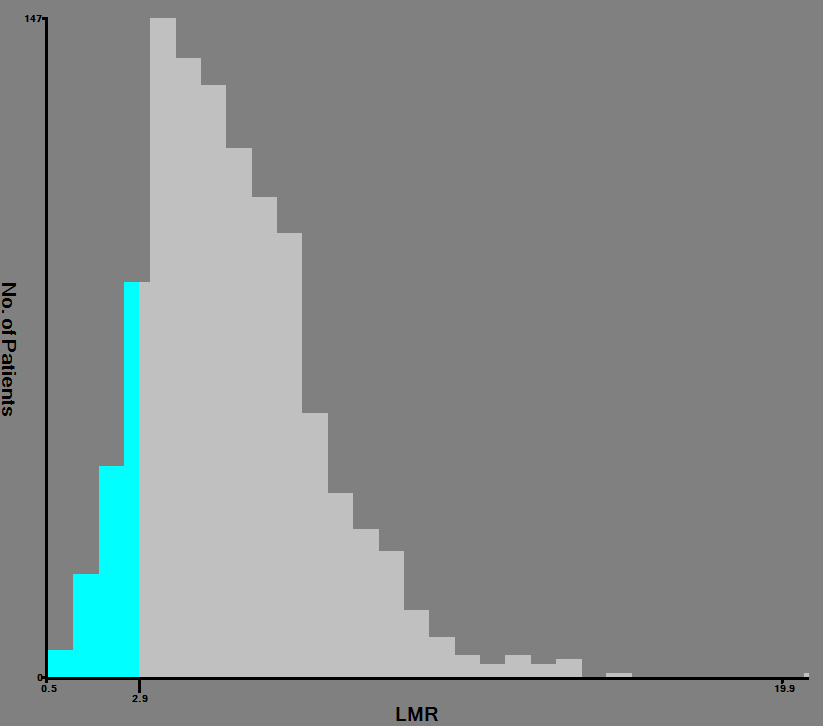** |
| **(D) PLR** |
| **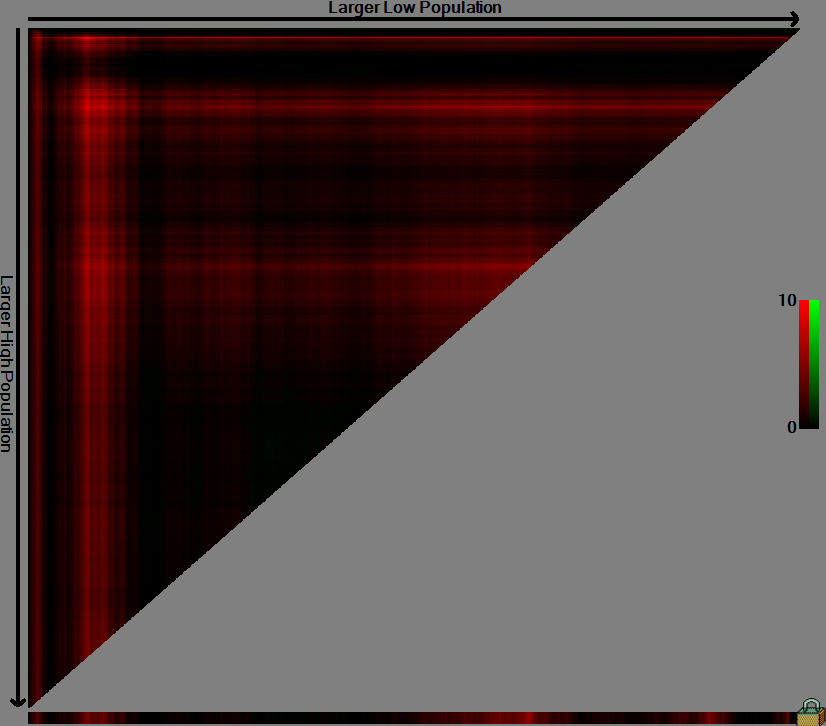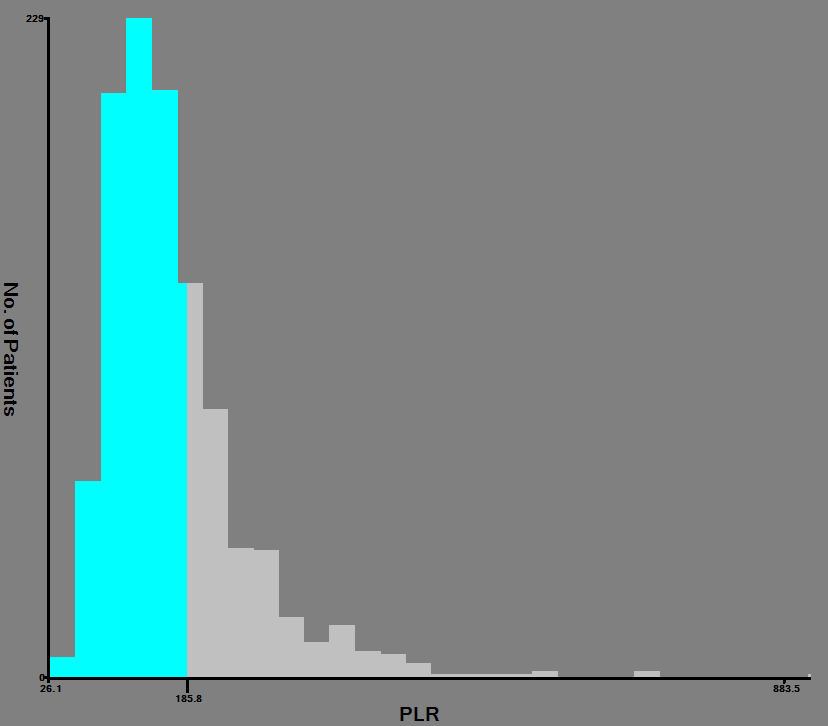** |

The points of the variable coloration of the X-tile plot represent the strength of the association at each division ranging from low (dark, black) to high (bright, red, or green). Red and green represent an inverse and direct association between the expression levels and survival of the variables respectively. The optimal cut-off value was defined as the values that produced the largest χ^2^ in the Mantel-Cox test, and these were set as 50.9 for PNI (A), 2.0 for NLR (B), 2.91 for LMR (C) and 185.84 for PLR (D) respectively.

**Supplementary Figure S3. Comparison of neutrophil-to-lymphocyte ratio (NLR), lymphocyte-to-monocyte ratio (LMR) and platelet-to-lymphocyte ratio (PLR) according to the CONUT.**

| **(A) NLR** | **(B) LMR** | **(C) PLR** |
| --- | --- | --- |
| 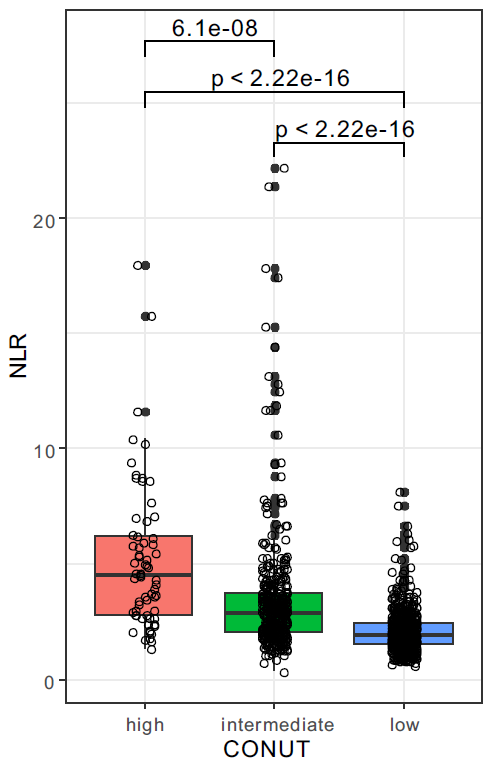 | 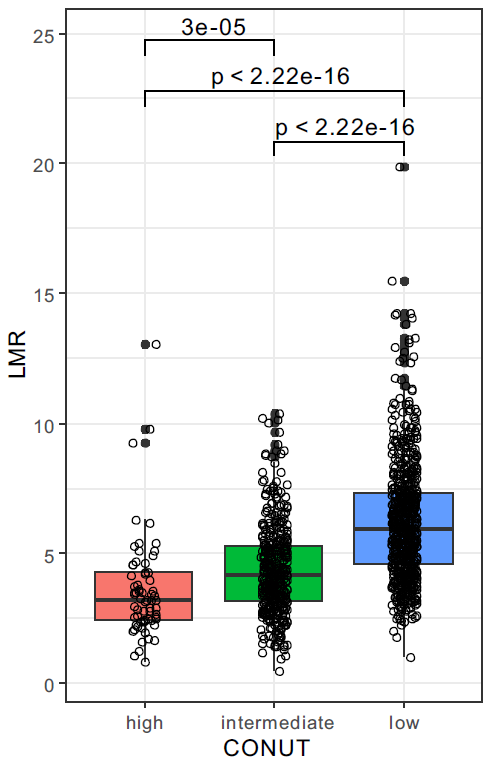 | 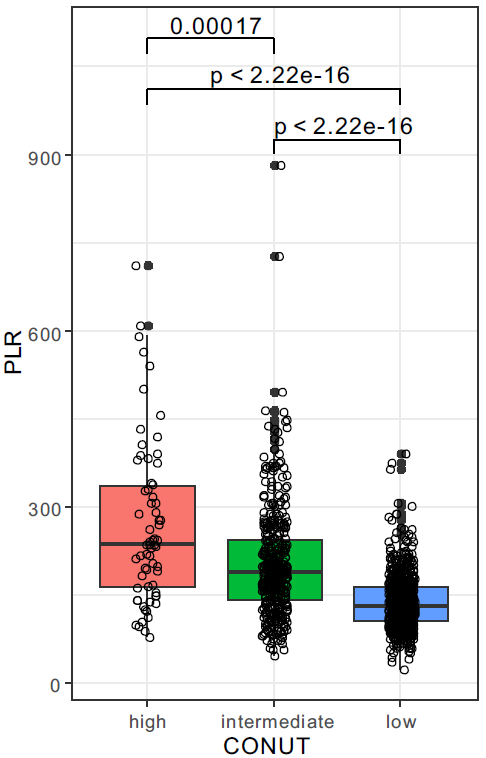 |


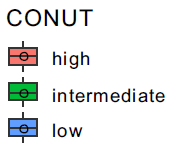


There were significant differences of median value of NLR (A) [4.515, (interquartile range, IQR) (2.803-6.183) in high vs. 2.860, IQR (2.060-3.690) in intermediate vs. 1.900, IQR (1.490-2.470) in low groups], LMR (B) [3.214, (IQR) (2.462-4.260) in high vs. 4.143, IQR (3.167-5.308) in intermediate vs. 5.970, IQR (4.564-7.308) in low groups], and PLR (C) [238.5, (IQR) (163.9-337.8) in high vs. 191.2, IQR (142.7-245.3) in intermediate vs. 133.8, IQR (105.9-165.1) in low groups] between CONUT score respectively (all *p*<.05).

**Supplementary Figure S4. Comparison of neutrophil-to-lymphocyte ratio (NLR), lymphocyte-to-monocyte ratio (LMR) and platelet-to-lymphocyte ratio (PLR) according to the PNI.**

| **(A) NLR** | **(B) LMR** | **(C) PLR** |
| --- | --- | --- |
| 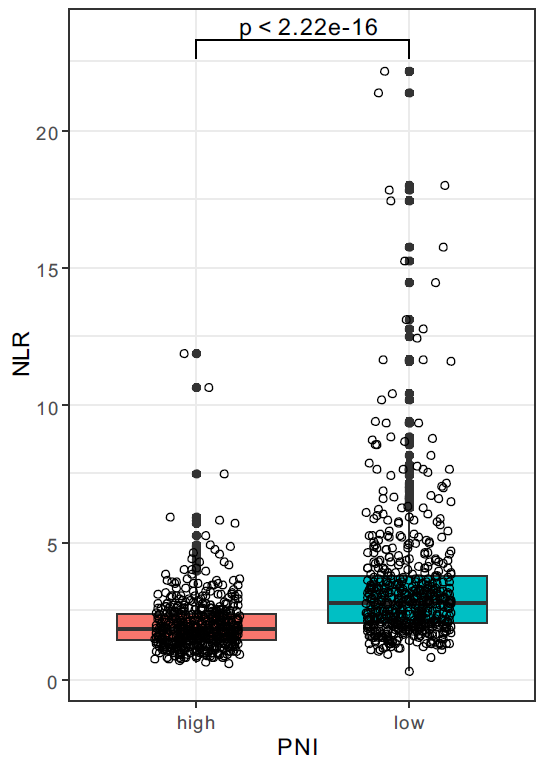 | 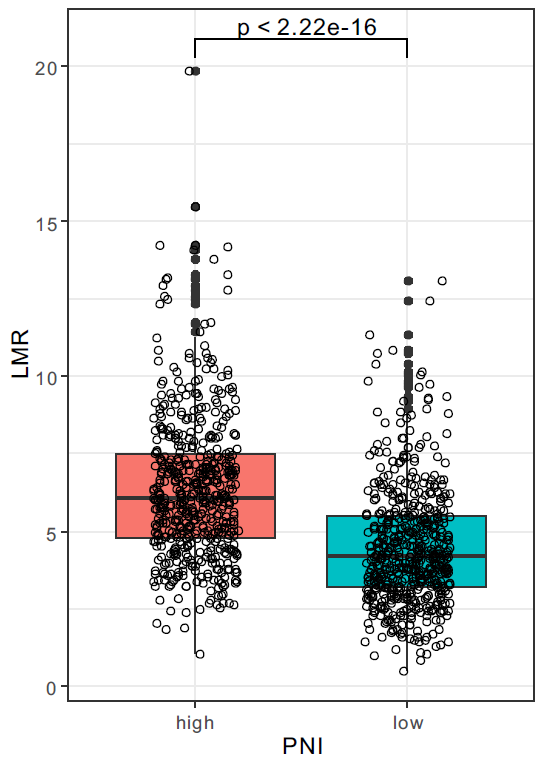 | 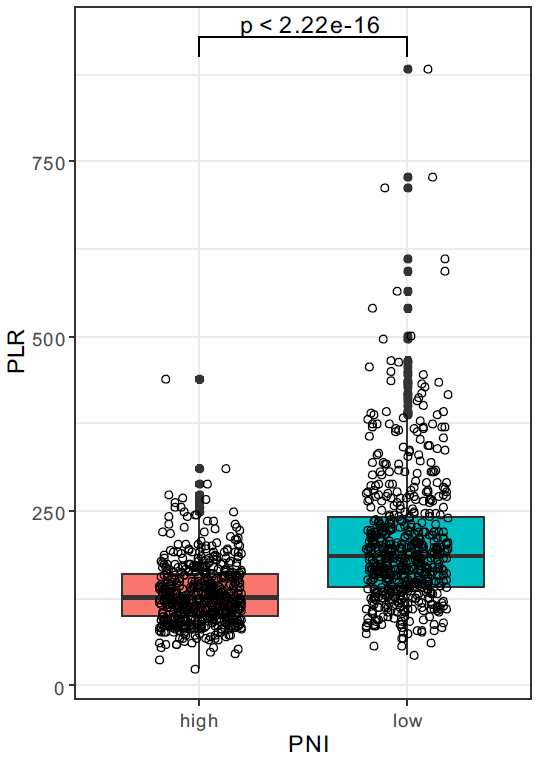 |

There were significant differences of median value of NLR (A) [1.82, (interquartile range, IQR) (1.43-2.40) in high vs. 2.78, IQR (2.03-3.72) in low groups], LMR (B) [6.074, IQR (4.804-7.455) in high vs. 4.167, IQR (3.182-5.462) in low groups] and PLR (C) [126.1, IQR (100.0-159.2) in high vs. 186.4, IQR (142.9-240.2) in low groups) between PNI respectively (all *p*<.05).

**Supplementary Figure S5. Comparison of Integrated AUC.**

| **(A) P-CONUT versus PNI** |
| --- |
| 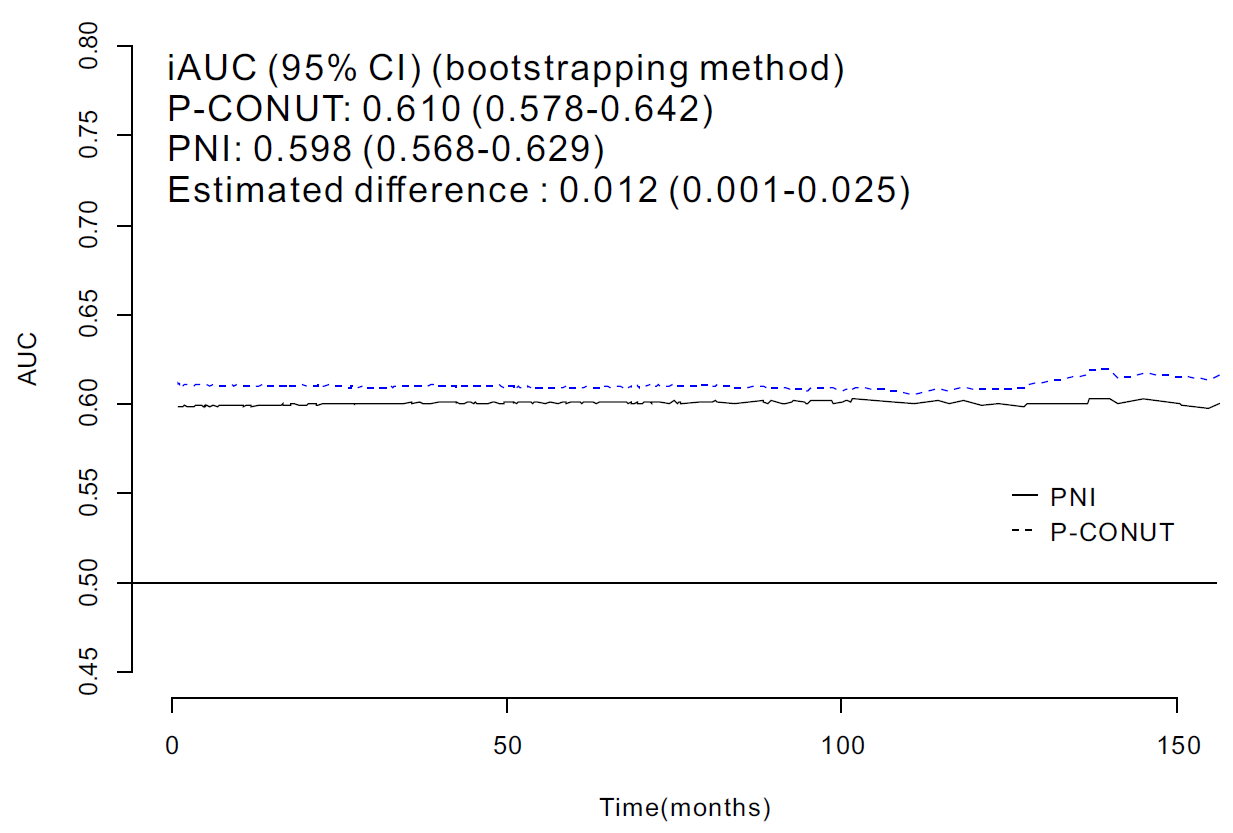 |
| **(B) P-CONUT versus CONUT** |
| 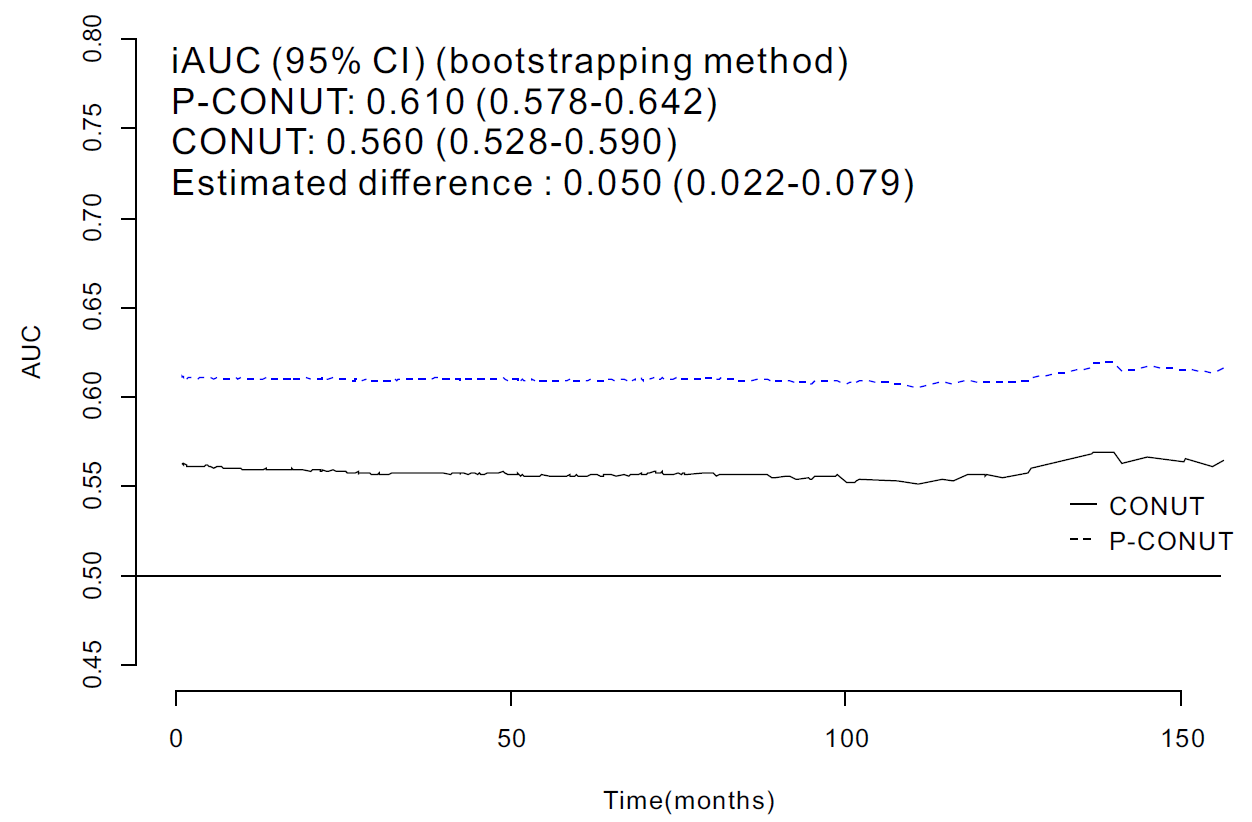 |

The iAUC of P-CONUT (0.610, CI: 0.578–0.642) was superior to those of the PNI alone (bootstrap iAUC mean difference=0.012; 95% CI=0.001–0.025) (A) and CONUT score alone (bootstrap iAUC mean difference=0.050; 95% CI=0.022–0.079) (B).

**Supplementary Table S2. Summary of prognostic impact of PNI in patients with colorectal cancer.**

| Author | Year/Nation | Stage | Number | Cut-off or stratification | Time of measurements | Out  come | HR (95% CI) or other outcomes | *p* | Comments |
| --- | --- | --- | --- | --- | --- | --- | --- | --- | --- |
| Mohri et al. ^1^ | 2013  /Japan | I-IV | 365 | ≤45 vs. >45 | Not mentioned | OS | 2.04  (1.39-3.01) | 0.0003 | Low PNI was associated with worse OS |
| Iseki et al.^2^ | 2015  /Japan | II-III | 204 | ≤40 vs. >40 | Within 2 weeks before the surgery | RFS | 1.011  (0.384-2.600) | 0.9825 | Low PNI was not associated with worse RFS and CSS |
|  |  |  |  |  |  | CSS | 1.119  (0.271-4.330) | 0.8700 |  |
| Tokunaga et al. ^3^ | 2015  /Japan | 0-III | 556 | ≤45.5 vs. >45.5 | Within 2 weeks before the surgery | OS | 3.98  (2.38-6.89) | <0.001 | Low PNI was associated with worse OS |
| Hayama et al.^4^ | 2020  /Japan | I-III | 336 | ≤40 vs. >40 | Not  mentioned | RFS | 1.240  (0.401-1.664) | 0.553 | Low PNI was not associated with worse RFS and OS |
|  |  |  |  |  |  | OS | 2.121  (0.883-6.481) | 0.139 |  |
| Tominaga et al.^5^ | 2020  /Japan | 0-III | 896 | ≤49.8 vs. >49.8 | Within 1 month before the surgery | OS | 2.133  (1.057-4.303) | 0.034 | Low PNI was associated with worse OS |
| Takamizawa et al.^6^ | 2020  /Japan | IV | 996 | ≤48.0 vs. >48.0 | 1^st^ visit or before initial treatment | OS | 1.39  (1.19-1.62) | <0.001 | Low PNI was associated with worse OS |
| Pian et al. ^7^ | 2021  /Korea | I (T1-2N0M0) | 305 | ≤49.3 vs. >49.3 | Not  mentioned | OS | 0.141  (0.054-0.371) | <0.001 | Low PNI was associated with worse OS and DFS |
|  |  |  |  |  |  | DFS | 0.105  (0.037-0.297) | <0.001 |  |
| Ahiko et al. ^8^ | 2021  /Japan | II-III | 1880 | ≤45 vs. >45 | Not  mentioned | OS | 1.54  (1.18-1.99) | 0.001 | Low PNI was associated with worse OS |

OS: Overall survival; RFS: Recurrence free survival; CSS: Cancer-specific survival; DFS: Disease-free survival

**Supplementary Table S3. Summary of prognostic impact of CONUT in patients with colorectal cancer.**

| Author | Year/  Nation | Stage | Number | Cut-off  or  stratification | Time of measurements | Out  come | HR (95% CI) or other outcomes | *p* | Comments |
| --- | --- | --- | --- | --- | --- | --- | --- | --- | --- |
| Iseki et al.^2^ | 2015  /Japan | II-III | 204 | ≥3 vs. <3 | Within 2 weeks before the surgery | RFS | 1.836  (0.844-3.713) | 0.1206 | Increased CONUT was associated with worse CSS, not RFS. |
|  |  |  |  |  |  | CSS | 1.853  (1.257-7.921) | 0.018 |  |
| Tokunaga et al. ^9^ | 2017  /Japan | I-III | 417 | 0-1  vs. 2-4  vs. ≥5 | Not mentioned | OS | 0-1 vs. 2-4:  2.74(1.30-5.87)  0-1 vs. ≥5:  5.92(2.3-14.92) | 0-1 vs. 2-4: 0.008  0-1 vs. ≥5: <0.001 | Increased CONUT was associated with worse OS. |
| Ahiko et al.^10^ | 2019  /Japan | I-IV | 830 | 0-1  vs. 2-3  vs. ≥4 | Not mentioned | OS | 0-1 vs. 2-3:  1.35(1.00-1.81)  0-1 vs. ≥4:  2.24(1.48-3.30) | 0-1 vs. 2-3: 0.048  0-1 vs. ≥4: <0.001 | Increased CONUT was associated with worse OS. |
| Hayama et al.^4^ | 2020  /Japan | I-III | 336 | ≥3 vs. <3 | Not mentioned | RFS | 1.797  (1.107-2.838) | 0.018 | Increased CONUT was associated with worse RFS and OS |
|  |  |  |  |  |  | OS | 2.53  (1.8-3.56) | <0.001 |  |
| Xie et al. ^11^ | 2020  /China | I-III | 512 | ≥1.5 vs. <1.5 | Not mentioned | DFS | 1.847  (1.339-2.548) | <0.001 | Increased CONUT was associated with worse DFS and OS |
|  |  |  |  |  |  | OS | 1.838  (1.317-2.564) | <0.001 |  |
| Takamizawa et al.^6^ | 2020  /Japan | IV | 996 | 0-1  vs. 2-3  vs. ≥4 | 1^st^ visit or before initial treatment | OS | 0-1 vs. 2-3 : 1.20(1.02-1.42)  0-1 vs. ≥4: 1.57(1.23-1.98) | 0-1 vs. 2-3: 0.032  0-1vs.≥4: <0.001 | Increased CONUT was associated with worse OS |
| Pian et al. ^7^ | 2021  /Korea | I (T1-2N0M0) | 305 | ≥3 vs. <3 | Not mentioned | OS | 2.393  (0.756-7.577) | 0.138 | Increased CONUT was not associated with worse DFS and OS |
|  |  |  |  |  |  | DFS | 2.893  (0.800-10.462) | 0.105 |  |
| Ahiko et al. ^8^ | 2021  /Japan | II-III | 1880 | 0  vs. 1-3  vs. ≥4 | Not mentioned | OS | 0 vs. 1-3: 1.31(1.01-1.71)  0-1 vs. ≥4: 1.67(1.08-2.59) | 0 vs. 1-3: 0.04  0-1 vs. ≥4: 0.02 | Increased CONUT was associated with worse OS. |

RFS: Recurrence free survival; CSS: Cancer-specific survival; OS: Overall survival; DFS: Disease-free survival

**References**

1. Mohri Y, Inoue Y, Tanaka K, Hiro J, Uchida K, Kusunoki M. Prognostic nutritional index predicts postoperative outcome in colorectal cancer. *World J Surg*. 2013;37(11):2688-2692. doi:10.1007/s00268-013-2156-9
2. Iseki Y, Shibutani M, Maeda K, et al. Impact of the Preoperative Controlling Nutritional Status (CONUT) Score on the Survival after Curative Surgery for Colorectal Cancer. *PLoS One*. 2015;10(7):e0132488. Published 2015 Jul 6. doi:10.1371/journal.pone.0132488
3. Tokunaga R, Sakamoto Y, Nakagawa S, et al. Prognostic Nutritional Index Predicts Severe Complications, Recurrence, and Poor Prognosis in Patients With Colorectal Cancer Undergoing Primary Tumor Resection. *Dis Colon Rectum*. 2015;58(11):1048-1057.
4. Hayama T, Ozawa T, Okada Y, et al. The pretreatment Controlling Nutritional Status (CONUT) score is an independent prognostic factor in patients undergoing resection for colorectal cancer. *Sci Rep*. 2020;10(1):13239. Published 2020 Aug 6. doi:10.1038/s41598-020-70252-2
5. Tominaga T, Nagasaki T, Akiyoshi T, et al. Prognostic nutritional index and postoperative outcomes in patients with colon cancer after laparoscopic surgery. *Surg Today*. 2020;50(12):1633-1643. doi:10.1007/s00595-020-02050-233951689
6. Takamizawa Y, Shida D, Boku N, et al. Nutritional and inflammatory measures predict survival of patients with stage IV colorectal cancer. *BMC Cancer*. 2020;20(1):1092. Published 2020 Nov 11. doi:10.1186/s12885-020-07560-3PMID: 31690275
7. Pian G, Oh SY. Comparison of nutritional and immunological scoring systems predicting prognosis in T1-2N0 colorectal cancer [published online ahead of print, 2021 Oct 7]. *Int J Colorectal Dis*. 2021;10.1007/s00384-021-04043-0. doi:10.1007/s00384-021-04043-0
8. Ahiko Y, Shida D, Nakamura Y, et al. Preoperative Nutritional Scores as Host-Related Prognostic Factors for Both Overall Survival and Postoperative Complications in Patients With Stage II to III Colorectal Cancer. *Dis Colon Rectum*. 2021;64(10):1222-1231. doi:10.1097/DCR.0000000000002033
9. Tokunaga R, Sakamoto Y, Nakagawa S, et al. CONUT: a novel independent predictive score for colorectal cancer patients undergoing potentially curative resection. *Int J Colorectal Dis*. 2017;32(1):99-106. doi:10.1007/s00384-016-2668-5
10. Ahiko Y, Shida D, Horie T, et al. Controlling nutritional status (CONUT) score as a preoperative risk assessment index for older patients with colorectal cancer. *BMC Cancer*. 2019;19(1):946. Published 2019 Nov 6. doi:10.1186/s12885-019-6218-8
11. Xie H, Nong C, Yuan G, et al. The value of preoperative controlling nutritional status score in evaluating short-term and long-term outcomes of patients with colorectal cancer following surgical resection. *J Cancer*. 2020;11(23):7045-7056. Published 2020 Oct 17. doi:10.7150/jca.49383
